# *Vibrio cholerae* O1 epidemic variants in Angola: a retrospective study between 1992 and 2006

**DOI:** 10.3389/fmicb.2013.00354

**Published:** 2013-11-28

**Authors:** Romy Valia, Elisa Taviani, Matteo Spagnoletti, Daniela Ceccarelli, Piero Cappuccinelli, Mauro M. Colombo

**Affiliations:** ^1^Dipartimento di Biologia e Biotecnologie C. Darwin, Università di Roma SapienzaRome, Italy; ^2^Centro de Biotecnologia, Universidade E. Mondlane MaputoMozambique; ^3^Department of Genetics, University College London Genetics Institute, Evolution and Environment, University College LondonLondon, UK; ^4^Department of Cell Biology and Molecular Genetics, Maryland Pathogen Research Institute, University of MarylandCollege Park, MD, USA; ^5^Dipartimento di Scienze Biomediche, Università di SassariSassari, Italy

**Keywords:** *V. cholerae* O1, Angola, mobilome, genomic islands, epidemic variants

## Abstract

Cholera is still a major public health concern in many African countries. In Angola, after a decade of absence, cholera reemerged in 1987, spreading throughout the country until 1996, with outbreaks recurring in a seasonal pattern. In 2006 Angola was hit by one of the most severe outbreaks of the last decade, with ca. 240,000 cases reported. We analyzed 21 clinical strains isolated between 1992 and 2006 from several provinces throughout the country: Benguela, Bengo, Luanda, Cuando Cubango, and Cabinda. We used two multiplex PCR assays to investigate discriminatory mobile genetic elements (MGE) [Integrative Conjugative Elements (ICEs), VSP-II, GI12, GI14, GI15, K, and TLC phages] and we compared the profiles obtained with those of different reference *V. cholerae* O1 variants (prototypical, altered, and hybrid), responsible for the ongoing 7th pandemic. We also tested the strains for the presence of specific VSP-II variants and for the presence of a genomic island (GI) (WASA-1), correlated with the transmission of seventh pandemic cholera from Africa to South America. Based on the presence/absence of the analyzed genetic elements, five novel profiles were detected in the epidemic strains circulating in the 1990s. The most frequent profiles, F and G, were characterized by the absence of ICEs and the three GIs tested, and the presence of GI WASA-1 and the WASA variant of the VSP-II island. Our results identified unexpected variability within the 1990s epidemic, showing different rearrangements in a dynamic part of the genome not present in the prototypical *V. cholerae* O1 N16961. Moreover the 2006 strains differed from the current pandemic *V. cholerae* O1 strain. Taken together, our results highlight the role of horizontal gene transfer (HGT) in diversifying the genetic background of *V. cholerae* within a single epidemic.

## Introduction

*Vibrio cholerae*, the etiological agent of cholera, a gastrointestinal infection, has been responsible for seven known pandemics with the seventh pandemic currently occurring. To date, over 200 distinct serogroups have been described, with only serogroups O1 and O139 associated with epidemic and pandemic cholera. *V. cholerae* serogroup O1 strains can be further classified in two biotypes, El Tor and Classical, based on differences in their phenotypic and genotypic traits (Kaper et al., 1995).

During the seventh pandemic several epidemic lineages of *V. cholerae* O1 El Tor have emerged. Most notable was the emergence, in 1992, of a new epidemic serogroup in India and Bangladesh, named O139 or Bengal, which initially displaced the local existing O1 El Tor strains (Faruque et al., 2003). Molecular analyses demonstrated horizontal gene transfer (HGT) as the mechanism by which *V. cholerae* O1 strains acquired the O139 surface antigen resulting in a new epidemic serogroup (Bik et al., 1995).

Subsequently, new variants of *V. cholerae* O1 with features of both the Classical and the El Tor biotypes have been repeatedly isolated in Asia and Africa and are collectively called “Atypical El Tor” strains. This group includes the Matlab types I, II, and III (Nair et al., 2002), Altered El Tor (Nair et al., 2006), Mozambique El Tor (Ansaruzzaman et al., 2004) and Hybrid El Tor strains (Safa et al., 2008).

Recent comparative genomics studies demonstrated that *V. cholerae* O1 strains circulating during the current seventh pandemic, which include prototypical El Tor strains, Atypical El Tor variants, and the O139 serogroup, belong to a single phyletic lineage named 7th pandemic clade (Chun et al., 2009). These variants are a result of HGT and slight divergence in strains from a common recent ancestor. Seventh pandemic El Tor strains are in fact characterized by a highly conserved genome background and different combinations of mobile genetic elements (MGE), referred to as the mobilome, such as Integrative Conjugative Elements (ICE), genomic islands (GIs), and prophages (Cho et al., 2010).

Seventh pandemic *V. cholerae* El Tor clones appear to have disseminated globally from a single source (the Bay of Bengal) in at least three independent waves, all of which reached Africa (Mutreja et al., 2011). Comparative genomic analyses have also suggested that the African continent served as a bridge for the spread of seventh pandemic *V. cholerae* strains from Asia to the Americas. Mutreja et al. (2011) observed that an Angolan strain, isolated in 1989, clustered at the base of the South American clade with a difference of only ten SNPs in the genomic backbone and the presence of two newly observed GIs: a novel variant of VSP-II, the VSP-II WASA (West African-South America), and a new GI named WASA-1 (Mutreja et al., 2011).

Angola is emerging from a 40 years war that ended in 2002 and resulted in significant social and economic disorders. Less than 4% of the population has access to potable water and adequate health and sanitary services. These conditions resulted in an almost immediate initiation of a cholera epidemic after *V. cholerae* isolates associated with the 7th cholera pandemic entered the African continent in the early 1970s. This region is now considered an endemic area for cholera (Lam et al., 2010). The most recent outbreak in Angola occurred from February to April 2013, and resulted in 610 cases and 8 deaths (International Society for Infectious Diseases, 2013).

Along with the most recent outbreak, two other major cholera epidemics were reported in Angola in the past 30 years. In April 1987 an epidemic started in the north-east of the country and rapidly spread to all 18 provinces, recurring in a seasonal pattern (Colombo et al., 1993). The outbreak was followed by a 10 years absence of the disease until 2006 when cholera re-emerged in Luanda and rapidly spread throughout the country. The 2006 outbreak was one of the most severe recent cholera epidemics in Africa (World Health Organization, 2007).

We previously demonstrated that the *V. cholerae* O1 strain responsible for the 2006 Angolan outbreak was an atypical O1 El Tor variant earlier detected only in Asia, and that it was clonally and genetically different from El Tor strains circulating in the 1990s in the same area (Ceccarelli et al., 2011a). Angolan strains from 2006 carried the SXT-related ICE*Vch*Ang3, associated with a more narrow multidrug resistance profile compared to the one conferred by plasmid p3iANG harbored by strains from the 1990s (Ceccarelli et al., 2006, 2011a). In light of the new insights into the phylogeny of seventh pandemic *V. cholerae* O1, we intended to reanalyze the Angolan epidemics in order to better understand the epidemiology of cholera in Angola and to confirm its role in bridging Africa and South America during waves of the seventh pandemic. In this study, we screened a collection of Angolan *V. cholerae* O1 isolates for the presence/absence of MGEs in order to study their collective mobilome and determine possible correlations with the known seventh pandemic variants (Spagnoletti et al., 2012).

## Materials and methods

### *V. cholerae* strains

Twenty-one clinical *V. cholerae* strains isolated during the two separate epidemics in Angola in 1996 and 2006 were analyzed (Ceccarelli et al., 2006, 2011a). Five completely sequenced reference strains were used as positive controls (Table 1): *V. cholerae* O1 N16961, isolated in India in 1975 (Kaper et al., 1995; Heidelberg et al., 2000); *V. cholerae* O139 MO10, isolated in India in 1992 (Ramamurthy et al., 1993); *V. cholerae* O1 CIRS101 (Altered El Tor), isolated in Bangladesh in 2002 (Nair et al., 2006); *V. cholerae* O1 B33 (Mozambique variant), isolated in Mozambique in 2004 (Ansaruzzaman et al., 2004); *V. cholerae* O1 MJ1236 (Matlab type I), isolated in Bangladesh in 1994 (Nair et al., 2002). *V. cholerae* strains analyzed in this work are listed in Table 1. Bacterial strains were routinely grown on LB Agar at 37°C for 16–18h with appropriate antibiotic selection and were maintained at −80°C in LB broth containing 15% (vol/vol) glycerol.

**Table 1:** ***V. cholerae* strains analyzed in this study**.

**Strain**	**Isolation**	**Multiplex 1**		**Multiplex 2**					**Profile**	**Wasa-1**
		**ICE**	**VSP-II^a^**	**phage K**	**GI-12**	**GI-14**	**GI-15**	**TLC**		
**REFERENCE**										
*V. cholerae* O1 N16961	1975, India	−	+	−	−	−	−	+	A	−
*V. cholerae* O1 CIRS101	2002, Bangladesh	ICE*Vch*Ind5	non−prototypical	−	−	−	−	+	B	−
*V. cholerae* O1 B33	2004, Mozambique	ICE*Vch*Moz10^b^	+	+	−	+	+	−	C	−
*V. cholerae* O1 MJ1236	1994, Bangladesh	ICE*Vch*Ban9^b^	+	+	+	+	−	−	D	−
*V. cholerae* O139 MO10	1992, India	SXT	+	+	−	−	−	+	E	−
*V. cholerae* O1 A5^c^	1989, Angola	−	WASA	n/a	n/a	n/a	n/a	n/a	n/a	+
**ANGOLA**										
*V. cholerae* O1 573	1992, Luanda	−	WASA	+	−	−	−	+	F	+
*V. cholerae* O1 588	1992, Luanda	−	WASA	+	−	−	−	+	F	+
*V. cholerae* O1 583	1992, Luanda	−	WASA	+	−	−	−	+	F	+
*V. cholerae* O1 611A	1992, Luanda	−	WASA	+	−	−	−	+	F	+
*V. cholerae* O1 582	1992, Luanda	−	WASA	−	−	−	−	+	G	+
*V. cholerae* O1 663	1993, Luanda	−	WASA	+	−	−	−	+	F	+
*V. cholerae* O1 1349	1994, Caxito (Bengo)	−	WASA	−	−	−	−	+	G	+
*V. cholerae* O1 1350	1994, Luanda	−	WASA	+	−	−	−	+	F	−
*V. cholerae* O1 1356	1994, Cuando Cubango	−	WASA	+	−	−	−	+	F	−
*V. cholerae* O1 1382	1994, Cuando Cubango	−	WASA	+	−	−	−	−	H	−
*V. cholerae* O1 819	1994, Dondo, Cuanza Norte	−	WASA	−	−	−	−	+	G	−
*V. cholerae* O1 1354	1994, Dondo, Cuanza Norte	−	WASA	−	−	−	−	+	G	+
*V. cholerae* O1 1383	1994, Benguela	−	WASA	+	−	−	−	+	F	−
*V. cholerae* O1 1357	1995, Cazenga, Luanda	−	WASA	+	−	−	−	+	F	+
*V. cholerae* O1 1359	1995, Sambizanga Luanda	−	WASA	+	−	−	−	+	F	+
*V. cholerae* O1 1358	1995, Tala Hady Luanda	−	WASA	−	−	−	−	−	I	+
*V. cholerae* O1 1360	1995, Cazenga, Luanda	−	WASA	−	−	−	−	−	I	−
*V. cholerae* O1 1361	1995, Cabinda	−	WASA	+	−	−	−	+	F	−
*V. cholerae* O1 908	1996, Cabinda	−	WASA	−	−	−	−	−	I	−
*V. cholerae* O1 VC175	2006, Luanda	ICE*Vch*Ang3	+	−	−	−	−	+	J	−
*V. cholerae* O1 VC189	2006, Luanda	ICE*Vch*Ang3	+	−	−	−	−	+	J	−

a VSP-II rearrangements were determined by either Multiplex 1 (VSPIIintF/R, VSPIIcutF/R) or VSP-II WASA (VSP2wasaF/R) PCR analysis (see Materials and Methods for further details).

b Sibling ICEs (Ceccarelli et al., 2011b).

c Information about this strain is derived from Mutreja et al. (2011).

### Primers and PCR conditions

Genomic DNA was prepared with a Wizard^®^ Genomic DNA Purification Kit (Promega), according to manufacturer's instructions. PCR was performed in 50 μl reaction mix containing 1 U of GoTaq DNA polymerase (Promega) and 50 ng/μL DNA. Amplification was performed in an automated thermocycler (BioRad MJ-Mini Personal Thermal Cycler). Oligonucleotides and PCR conditions for the double multiplex PCR were optimized as previously described (Spagnoletti et al., 2012).

WASA-1 and VSP-2 WASA presence was investigated with new primer pairs designed using Angolan strain *V. cholerae* O1 El Tor A5 (Mutreja et al., 2011) as reference (Accession no ERS013245): (i) wasa1F (5′-CCAAAGCAGAGAGACGCA-3)′, and wasa1R (5′-GTTCTCACCTTCTTCCGCA-3′) giving an amplicon size of 464 bp; (ii) VSP2wasaF (5′-GTGCTGTATTTGGTTTGATGGGT-3′), and VSP2wasaR (5′-GATAGTGGTTTCGCTGAGATTGT-3′), resulting in an amplicon size of 438 bp. Oligonucleotides were obtained from PRIMM srl (Milano, Italy).

## Results

**Figure 1:**
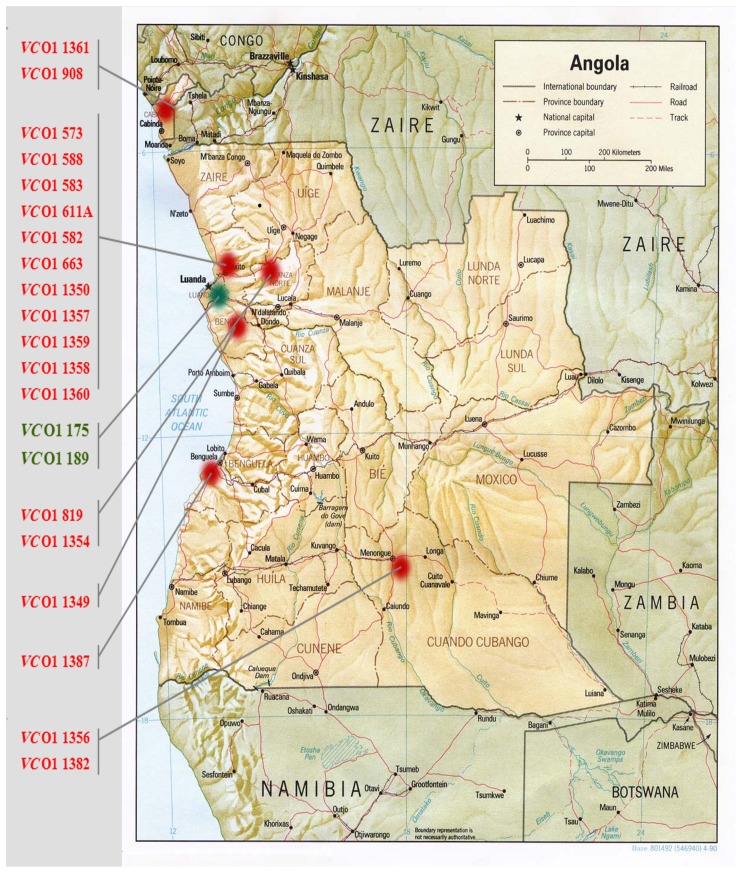
**Map of Angola showing the locations of *V. cholerae* O1 isolation in this analysis.** Strains isolated during the 1992–1996 epidemics are indicated in red, strains isolated during 2006 epidemics are indicated in green.

Of the 21 strains analyzed during this study, 19 were isolated between 1992 and 1996 from the provinces of Benguela, Bengo, Luanda, Cuando Cubango (South-east), and the Cabinda enclave (North-west) (Figure 1). Two clinical strains isolated in Luanda in 2006 were also included in this study. All the strains were subjected to the double multiplex PCR screening and their amplicon profiles compared with the reference strains (Table 1, profiles A–J). WASA-1 analysis was not included in the profile definition but was analyzed separately to be consistent with the original double Multiplex PCRs approach (Spagnoletti et al., 2012). It is important to note that primers included in Multiplex 1 (VSPIIintF/R and VSPIIcutF/R) and used to discriminate prototypical VSP-II from other variants (Spagnoletti et al., 2012) gave no amplification for both the deleted CIRS-like VSP-II island and the WASA VSP-II. Therefore, rearrangements in the non-prototypical VSP-II islands were further tested with the VSP-2wasaF/R primer pair. The five reference strains showed unique expected profiles (Spagnoletti et al., 2012), as listed in Table 1.

### Analysis of *V. cholerae* O1 epidemic strains isolated between 1992 and 1996

Six clinical strains isolated in 1992 and 1993 in Luanda showed two different mobilome profiles. Profile F (four strains from 1992 and one strain from 1993) is characterized by the presence of a non-prototypical VSP-II, and phages K and TLC; and profile G (strain 582 from 1992) carries the non-prototypical VSP-II and the TLC phage. All non-prototypical VSP-II islands were confirmed as carrying WASA insertion. Additionally, all six strains showed the expected amplification for WASA-1 GI.

Seven clinical *V. cholerae* O1 strains isolated in five different provinces of the country in 1994 (Figure 1) gave three profiles (Table 1). Similar to the 1992 and 1993 strains isolated from Luanda, the two most common profiles were F and G. Profile F strains were isolated from Luanda (isolate 1350), Cuando Cubango (isolate 1356), and Benguela (isolate 1383). Profile G strains were isolated from Bengo province (isolate 1349), and Dondo (Cuanza Norte) (isolates 819 and 1354). A previously unobserved profile H (non-prototypical VSP-II and phage K) was observed in strain 1382 isolated in Cuando Cubango. All non-prototypical VSP-II islands were confirmed as carrying the WASA insertion. *V. cholerae* strains 1349 and 1354 gave a positive amplification for WASA-1 GI while the remaining five strains gave negative amplification for this cluster.

Five clinical *V. cholerae* O1 strains from 1995 and one from 1996 were isolated in the provinces of Luanda and Cabinda (Figure 1). Strains isolated in four different municipalities of the Luanda province in 1995 showed two profiles: profile F (strains 1357 and 1359), and profile I (strains 1358 and 1360) in which the sole non-prototypical variant of VSP-II was amplified. The two strains isolated in the Cabinda enclave showed profiles F and I in 1995 (strain 1361) and 1996 (strain 908), respectively. All non-prototypical VSP-II islands were confirmed as carrying the WASA insertion. *V. cholerae* strains 1357, 1358, and 1359 from Luanda gave a positive amplification for the WASA-1 GI while the remaining 3 strains (isolates 1360, 1361, and 908 from Luanda and the Cabinda enclave, respectively) were negative for this cluster.

### Analysis of *V. cholerae* O1 epidemic strains isolated in 2006

As anticipated by our previous study (Ceccarelli et al., 2011a), the two *V. cholerae* O1 strains isolated in Luanda during the 2006 epidemic, shared a common and previously unobserved mobilome profile J (Table 1). These strains harbored the prototypical intact variant of VSP-II, an ICE element (ICE*Vch*Ang3, sibling of ICE*Vch*Ind5), and TLC. Both were negative for the presence of WASA-1 and VSP-II WASA genetic clusters.

## Discussion

This retrospective study is based on a collection of *Vibrio cholerae* O1 clinical strains, isolated from 1992 to 2006 from different provinces in Angola (Figure 1 and Table 1). Despite encouragement from local national health authorities, it was not possible to isolate strains over a broader temporal and geographic scale due to security and logistic constraints caused by socio-economic conditions. Thus, we are aware of the potential spatial and temporal biases inherent in this set of strains. Half of our isolates were recovered from cases in Luanda, and all but two isolates were recovered between 1992 and 1996. Nevertheless, we believe the strains presented in this study represent an important source of data depicting the presence of a variable population of *V. cholerae* O1 strains in the country.

The epidemiology of *V. cholerae* O1 in Africa can be explained by multiple introductions of cholera from endemic regions of Asia, with the first introduction following the early dissemination of seventh pandemic in the 1970s (Kaper et al., 1995). In our previous analysis we hypothesized that *V. cholerae* O1 strains responsible for the first epidemic (1987–1996) in Angola are progeny of the prototypical strain represented by *V. cholerae* O1 El Tor N16961, with the only difference being the presence of the p3iANG plasmid (Ceccarelli et al., 2011a). However, our current study revealed that all *V. cholerae* O1 strains from 1987 to 1996 harbored the WASA variant of the VSP-II GI, and 10 of these carried also the WASA-1 GI. Therefore, these strains differed in MGE content from *V. cholerae* N16961 (profile A). An even greater level of diversity was identified in these strains when tested for the presence of the K and TLC phages. These analyses resulted in the assignment of four distinct profiles (F–I) among strains isolated between 1987 and 1996 in Angola.

The two strains isolated in 2006 showed a mobilome profile highly similar to that of the altered El Tor *V. cholerae* O1 strain CIRS101, including the presence of ICE*Vch*Ang3, an ICE element of the SXT/R391 family sibling with ICE*Vch*Ind5, the most widespread ICE present in *V. cholerae* O1 CIRS101-like strains isolated in the Indian Subcontinent and worldwide (Ceccarelli et al., 2011a,b,c). Again, significant differences between the 2006 Angolan strains and *V. cholerae* CIRS101 were identified in the VSP-II island. The 2006 Angolan strains encoded the prototypical VSP-II, while many currently isolated pandemic *V. cholerae* O1 CIRS101-like strains isolated worldwide after 2001 encode a deletion in VSP-II (Taviani et al., 2010). These results suggest that epidemic strains circulating in Angola in 2006 harbored a genetic background with features similar to strains responsible for the first (profile A) and the third (profile B) pandemic waves.

All strains in this analysis were negative for GIs-12, −14, and −15, which are specific GIs of atypical El Tor strains similar to *V. cholerae* O1 MJ1236 and B33 (Grim et al., 2010). The latter was isolated in Beira, Mozambique, in 2004, but it was recently demonstrated to be present in Austral Africa since the 1990s (Spagnoletti et al., 2012). By testing these three GIs as molecular markers, our analysis suggests that the Angolan epidemics were not directly influenced by contemporary eastern Africa epidemics, which are thought to have been initiated by the second pandemic wave (Mutreja et al., 2011).

Our analysis revealed the presence of the WASA-1 GI in all *V. cholerae* O1 strains from 1992, whereas the same element was absent in recently recovered isolates. The VSP-II WASA variant was instead present in all *V. cholerae* O1 strains. The presence of the WASA clusters in Angolan *V. cholerae* O1 strains confirmed the Mutreja's hypothesis, based only on one isolate from 1989, that the transmission of the seventh pandemic to South America may have occurred via the African continent and, specifically, through Angola (Mutreja et al., 2011). Furthermore, it suggests that transmission of a strain which encoded both VSP-II-WASA and WASA-1 is likely to have occurred during the 1980s, as previously reported (Lam et al., 2010; Mutreja et al., 2011).

In conclusion, the history of the Angolan epidemic adds new and valuable information to evolutionary history of *V. cholerae* O1 El Tor within the seventh pandemic. At the time-scale presented here and elsewhere, this evolution occurs mainly via lateral gene transfer events driven by environmental factors. Interestingly, none of the Angolan strains showed a mobilome profile similar to those of the representative 7th pandemic strains. The variability within these strains was greater than expected, as strains isolated within the same epidemic were shown to harbor different constellations of mobile elements. A similar variability was previously observed in a collection of strains isolated in Mozambique in 1997–1998 (Spagnoletti et al., 2012), thus reflecting frequent HGT dynamics similar to the Angolan epidemic.

### Conflict of interest statement

The authors declare that the research was conducted in the absence of any commercial or financial relationships that could be construed as a potential conflict of interest.
